# Complement Component C1q: Historical Perspective of a Functionally Versatile, and Structurally Unusual, Serum Protein

**DOI:** 10.3389/fimmu.2018.00764

**Published:** 2018-04-10

**Authors:** Kenneth B. M. Reid

**Affiliations:** Green Templeton College, University of Oxford, Oxford, United Kingdom

**Keywords:** C1 activation, C1q, collagen-like structure, globular heads, tumor, aging

## Abstract

Complement component C1q plays an important recognition role in adaptive, and innate, immunity through its ability to interact, *via* its six globular head regions, with both immunoglobulin and non-immunoglobulin activators of the complement system, and also in the clearance of cell debris, and by playing a role in regulation of cellular events by interacting with a wide range of cell surface molecules. The presence of collagen-like triple-helical structures within C1q appears crucial to the presentation, and multivalent binding, of the globular heads of C1q to targets, and also to its association with the proenzyme complex of C1r_2_–C1s_2_, to yield the C1 complex. The possible role that movement of these collagen-like structures may play in the activation of the C1 complex is a controversial area, with there still being no definitive answer as to how the first C1r proenzyme molecule becomes activated within the C1 complex, thus allowing it to activate proenzyme C1s, and initiate and the consequent cascade of events in the activation of the classical pathway of complement. The globular heads of C1q are similar to domains found within the tumor necrosis factor (TNF) superfamily of proteins, and have been shown to bind to a very wide range of ligands. In addition to its well-defined roles in infection and immunity, a variety of other functions associated with C1q include possible roles, in the development of problems in the central nervous system, which occur with aging, and perhaps in the regulation of tumor growth.

## Introduction

Prior to the formal proof of there being collagen triple helical coils present in the C1q molecule (1), it was considered that any such protein, containing that feature, would most likely play a structural role in the extracellular matrix, rather than being involved in the activation of the serum complement system. However, it was recognized by the end of the 1980s (2) that several other serum proteins, besides subcomponent C1q, also contained collagen-like regions, and were likely to be involved in innate immune effector systems. These included the C-type lectins, mannose binding lectin (MBL), bovine conglutinin, and lung surfactant protein A. Indeed, conglutinin was the first vertebrate lectin to be characterized by virtue of its function to promote the agglutination of erythrocytes coated with activated complement components (3). It is now known that there are several other lectins, such as lung surfactant protein D, the ficolins, bovine serum lectin (CL-43), in a growing family of proteins, containing collagen-like regions, which are involved in immune defense. These proteins display a wide range of binding properties, *via* both their globular head regions and collagen-like triple-helical regions, toward both immune targets and cell surface receptors, and participate as a bridge between innate and adaptive immunity. The relationship between structure and function with respect to the many binding, and triggering, properties shown by C1q should now be able to be even more fully explored, by generation of point mutation variants, as a result of the major achievement of expression of the functionally fully active recombinant form of this structurally complicated protein, composed of three different polypeptide chains, in a mammalian cell system (4).

## Early Characterisation of C1q and Determination of Its Structure

The C1q protein was first, accurately, described, in 1961, as a “11s thermolabile serum protein which precipitates γ-globulin aggregates and participates in immune hemolysis” (5), thus highlighting interesting features about its large size (460 kDa) and its binding properties (to immunoglobulin complexes) and its function (participation in complement-mediated hemolysis of antibody-coated red cells). In 1963 (6), Lepow et al. showed that the euglobulin fraction of human serum (proteins precipitated in low ionic strength buffer, at pH 5.5), which contained the then defined C1component of the complement system, could be fractionated, by ion-exchange chromatography into three subcomponents, which were defined, based on their elution positions, from an ion-exchange column, as C1q, C1r, and C1s (the nomenclature a, b, and c…, was not used, in order to avoid confusion with “C1a” being used for activated C1, at that time). It was shown that all three subcomponents were required to reconstitute the original C1 hemolytic activity. The use of further, new at the time, techniques, such as gel-filtration and affinity chromatography, allowed the isolation of highly purified C1q to perform detailed structural and functional studies.

Early chemical studies of human and rabbit C1q (7–9) provided indirect evidence, that there may be collagen-like structures within C1q, since it was reported to have an unusually high glycine content, to contain hydroxylysine and hydroxyproline residues and disaccharide units of glucosylgalactose, linked to the hyroxylysine, and it had a great susceptibility to collagenase. The first direct evidence for the presence of collagen-like amino acid sequence, in the A-chain of C1q, was obtained in 1974 (10).

It was then shown that the preparation of the collagen-like regions of C1q could be achieved by limited proteolysis of the native intact molecule with pepsin at pH 4.45, when the globular head regions are digested to small peptides leaving the large, 190 kDa, collagen-like region intact (1, 11). When viewed in the electron microscope, C1q was seen to be composed of six peripheral globular “head” regions, which are each joined by a collagen-like connecting strand to a fibril-like central portion/stalk (1, 12, 13).

**Figure 1:**
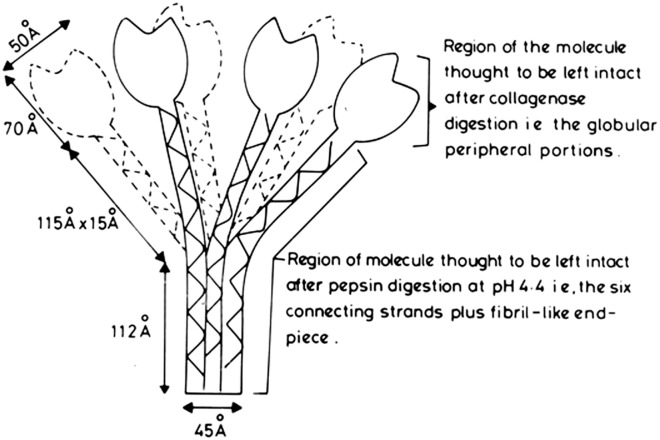
Proposed model of human subcomponent C1q. Initial diagram, drawn up in 1975, of the first published molecular model proposed for C1q (14). It was based on the electron microscopy measurements (12, 13), the amino acid sequencing and physical chemistry results of the studies on the 190 kDa pepsin-resistant fragment of C1q (1, 11), and the assumption that the collagen-like regions in the A-, B-, and C-chains of C1q form a triple helical collagen-type structure (denoted by the solid, broken, and wavy lines), and the C-terminal approximately 140 amino acid residues, in each of the A-, B-, and C-chains, form a globular heterotrimeric structure of 47.8 kDa (which should, more correctly, be shown as globular units, rather than the tulip-flower shapes as shown in the initial diagram). From the dimensions shown, which are averages of those given in the electron microscopy studies (12), the following comparisons can be made: length of collagen-like fiber plus fibril-like end piece = 115 + 112 = 227 Å. Length of triple helix proposed from amino acid sequences = 78 × 2.9 = 226 Å (2.9 Å is the rise of collagen helix per residue, and there are 78 residues, over the collagen-like regions, in each of the three chains of C1q).

All these studies allowed the proposal in 1976 (14) of a molecular model for subcomponent C1q (Figure 1), which has stood the test of time, in which there are 18 polypeptide chains (6 A-, 6 B-, and 6 C-chains), with disulfide bonds between the A- and B- chains and between pairs of C-chains, thus yielding nine dimers, i.e., six A–B dimers and three C–C dimers. The complete derived amino acid sequence, along with the characterization and organization of the genes encoding all three polypeptide chains of C1q, was completed in 1991 (15).

The use of large fragments of C1q, produced by limited proteolysis with either collagenase or pepsin, allowed direct analysis of the primary functions of the two, very different, regions of the C1q molecule. The globular head regions of C1q can be prepared by digestion with collagenase at pH 7.4, which results in a rapid loss of C1q function (9, 16). These preparations, of globular subunits, with a molecular weight of 47 kDa, were shown (16) to be able to inhibit the binding of C1q to IgG immune complexes, thus directly illustrating the role of the peripheral globular head units in binding to IgG. The determination of the crystal structure globular head region of C1q (17), also prepared by collagenase digestion, has shown that it is an almost spherical heterotrimeric assembly (formed from the C-terminals regions of the A-, B-, and C-chains). This allowed molecular modeling with two of its well-defined targets, IgG and C-reactive protein (CRP), providing a good illustration of the versatility in binding shown by the globular heads of C1q. It was found that the large pepsin-resistant fragment of C1q, composed almost entirely of the collagen-like regions of the molecule, could act as an effective inhibitor of the reconstitution of whole C1 hemolytic activity, when intact C1q was mixed with C1r_2_–C1s_2_ (18). It has also been found that the pepsin-resistant fragment of C1q bound the unactivated C1r_2_–C1s_2_ with approximately the same strength as that of the intact C1q molecule (19). These studies provided strong functional evidence that the C1r_2_–C1s_2_ interacted primarily with the collagen-like regions of C1q, and was in agreement with electron microscopy studies of the whole C1 complex (20) which suggested that the C1r_2_–C1s_2_ binding site is in the middle of the triple helical collagen-like regions, close to where the collagen-like strands diverge out from the central fibril-like region (Figure 1).

## Structural Model of the Collagen-Like Region of C1q, Its Interaction with C1r_2_–C1s_2_ and Possible Involvement in Activation of the C1 Complex

It is clear from electron microscopy, and all the protein structural data available, that the 460 kDa C1q molecule adopts a bouquet of flowers shape, comprising six heterotrimeric collagen-like triple helices that associate in their N-terminal half to form a fibril-like structure, then diverge at a bend, or “kink,” approximately half-way along the collagen-like region to form six individual “stalks,” each terminating in a C-terminal heterotrimeric globular domain (Figure 1). Other serum proteins (MBLs, Ficolins) have an overall structural similarity to C1q, and also interact with their associated proteases *via* their collagen-like regions. It was noted that, on alignment of all the known, collagen-like sequences, present in various chains of C1q, MBL, and Ficolin, from several species, that there is a conserved amino acid sequence (-Hyp-Gly-Lys-Xaa-Gly-Pro-) in which it was shown, by site-directed mutagenesis of Ficolin A, that the lysine is the critical residue involved in interaction with its associated protease, MASP (21). This conserved site, also present in each of the three chains of C1q, is six Gly-Xaa-Yaa- triplets C-terminal to the link region of C1q, and it was, therefore, postulated that this is likely to be the major binding site for C1r_2_–C1s_2_ on the stalks of C1q (21). The importance of these lysine residues, in the B- and C- chains, and lesser extent the A-chain, for the interaction, and activation, of C1r_2_–C1s_2_ was elegantly formally proved by their mutation to alanine residues, with consequent loss-of-function. Further studies, involving the determination of the crystal structure of the CUB1-EGF-CUB2 region of C1s bound to a short triple helical collagen-like peptide, containing the important conserved lysine, provided strong direct structural evidence for the proposed, precise interaction site between C1q and C1r_2_–C1s_2_ (22).

## C1q Targets and Receptors

The structural studies on globular head region of C1q show that it is member of the growing TNF superfamily (17, 23) and functional studies indicate that the C1q heads can bind a wide range of self and non-self ligands.

Primary targets for the six globular heads of C1q are the multiple Fc regions presented within immune complexes, containing IgG or IgM antibodies. Well-defined non-immunoglobulin targets, for the C1q heads, include CRP (24) and pentraxin 3, as well as lipopolysaccharides and bacterial porins. Apoptotic cells also form a major target (25–28), probably *via* C1q binding to phosphatidylserine and double-stranded DNA, thus allowing for opsonization and effective phagocytosis of cell debris (28, 29), and enhancement of the immunosuppressive nature of the apoptotic cells.

One of the first molecules to be proposed as a receptor, for the globular heads of C1q, is gC1qR/p33 (30), but this molecule, although found at the surface of a wide variety of cells, has been shown to be present mainly in mitochondria. Since it does not possess a transmembrane domain, or a lipid anchor, it appears that it must always have to interact with other cell surface molecules, in order to modulate intracellular functions.

After activation of the C1 complex, control of the activated C1r and C1s is mediated by C1-inhibitor which forms covalent complexes with both the activated C1r and C1s, rapidly removing them from the C1 complex, leaving the entire collagen-like region of C1q free to interact with potential cell-surface receptors. One such putative receptor was a 60 kDa molecule isolated from Raji cell membranes (31) and to bind to the collagen region of C1q (and thus became to be defined as cC1qR). The NH2-terminal amino acid sequence of this molecule, isolated from endothelial cells (32) was found to be identical to that of C1qR, isolated from tonsil lymphocytes, and also to that of calreticulin (33) The C1qR preparation was shown to bind to the collagen-like regions of C1q and several other collagen-like proteins (34). Although cC1qR/C1qR/calreticulin can be found at the surfaces of many cell types, it is primarily found in the endoplasmic reticulum, and lacks a transmembrane domain. Thus care must be taken to define both gC1qR and cC1qR as “C1q-binding proteins,” rather than as true “transmembrane receptors.”

A wide range of other cell surface molecules have now been shown to interact with the either the heads, or collagen-like regions, of C1q, and occasionally with both. This area has recently been thoroughly and critically reviewed (35). The putative receptors were neatly placed in four main groups, based on the nature of their extracellular domains: (i) large multi-modular ectodomains involved in interaction with multiple ligands (CR1, LRP1, and the scavenger receptors SR-F1 and SR-F3); (ii) integrins (α2-β1 and CR3/αM-β2); (iii) Ig-like receptors (RAGE, LAIR-1, and CD33); and (iv) the C-type lectin receptors, DC-SIGN and DC-SIGNR.

## Current Views as to the Mechanism of Activation of the C1 Complex

There are two recent publications (36, 37), which address the possible mechanism by which it is considered the first steps of activation of proenzyme C1r, within the C1 complex, may take place, and they come to significantly different conclusions.

One view, the intramolecular view (36), is that the proenzyme C1r domains must be linked together at the center of the heterotetramer C1r_2_–C1s_2_, and that autoactivation of proenzyme C1r occurs as soon as the contacts between the catalytic domains are broken, possibly by flexibility of the collagen-like stems of C1q, on contact of the globular heads with targets. These conclusions derive from previous observations and are associated with studies on the interactions, in solution, of fragments (the CUB1-EGF-CUB2 fragments) of the C1r and C1s involved in binding to C1q, and also the crystal structure of a complex of these two fragments, allowing a close analysis of the C1s–C1r interface.

The other view, the intermolecular view (37), is that the C1 complex cross-activates by interacting with neighboring C1 complexes. This model is based on synchrotron small-angle X-ray scattering and electron microscopy studies and concludes that there is cleavage of proenzyme C1r in one C1 complex by C1r in a neighboring complex. The two models differ in the precise manner in which the C1r_2_–C1s_2_ complex is aligned, at the now generally agreed position, within the collagenous stems of C1q. The intramolecular view (36) does appear to be consistent with the first-order kinetics, reported by several groups, as regards activation of C1, but does rely upon contacts between the catalytic domains being broken, presumably by flexibility within the collagen-like stems on binding to a target, and this has not yet been formally proven. However, although collagen-like structures are seen as structures with only limited flexibility, all observations, made on C1q, suggest flexible hinge movements at the level of the “kink,” which can modulate the positions of the six globular domains. The intermolecular model (37), in which the serine protease domains are considered to be located at the periphery of the C1r_2_–C1s_2_ complex is consistent with the fact that C1 can bind to a structurally diverse range of activators and allows intermolecular activation between neighboring complexes. In a very recent study (38), cryo-electron microscopy was used to examine C1 bound to monoclonal antibodies, and the authors observed heterogeneous structures of single and clustered C1-IgG1 hexamer complexes. This structural data was interpreted as showing that, upon antibody binding, the C1q arms condense, thus inducing rearrangements of the C1r_2_–C1s_2_ complex and tilting the C1q’s cone-shaped stalk. Thus, it was concluded that C1r perhaps could activate C1s within single, strained C1 complexes, or between neighboring C1 complexes on surfaces (38).

Final general acceptance of one, or other, or indeed a combination, of these models of C1 activation awaits further study.

## Biosynthesis of C1q, Levels of Serum C1q, and Deficiency of C1q

Unlike most of the other complement proteins, which are mainly liver-derived, C1q is synthesized primarily by macrophages, as demonstrated by the fact that bone marrow transplantation from wild-type mice into C1q-deficient (C1qa^−/−^) mice was able to restore the normal serum levels of C1q (39). This finding has prompted the use of hematopoietic stem cell transplantation in the remarkable, and successful, treatment of genetic human C1q deficiency (40), a condition where the complete absence of C1q function results in an exceptionally high risk of severe lupus erythematosus, and complications with skin and renal diseases.

In normal healthy human sera, C1q has a concentration of around 80 µg/ml (0.17 µM in serum) and thus is present in an approximately equimolar concentration to that of the C1r_2_–C1s_2_ complex (50 µg/ml of each of C1r and C1s, thus 0.15 µM C1r_2_–C1s_2_). The concentration of C1q rises quite steeply with aging, reaching 161 µg/ml, in the 60–81, years old, age group (41). It is of interest that the high serum level of C1q could surprisingly be reduced by a simple exercise regime, down to almost normal levels, which may be of significance to health in old age. An even more dramatic (10- to 300-fold) increase in C1q levels in the central nervous system (42) has been reported in mouse and human brains, with the highest levels being seen in close proximity to synapses and central regions of the brain. Interestingly, aged C1q-deficient (C1qa^−/−^) mice showed less cognitive and memory decline in hippocampus-dependent behavior tests compared to their wild-type litter mates (42), thus suggesting that C1q may play a role in the development of problems, during aging, that are seen in the central nervous system.

Another connection between C1q and aging has emerged from studies that there is elevated Wnt signaling in aging mice, where muscle stem cells have an increased tendency to fibroblastic differentiation (43). This appears to be due to the binding by one, or more, serum factors, to the Frizzled family of cell surface receptors, thus causing Wnt receptor signaling, and one Frizzled-binding protein has been identified to be C1q (44). It has been proposed that when C1q, within the C1 complex, binds to Frizzled then activated C1s cleaves lipoprotein receptor 6, which is a Wnt co-receptor, thus causing canonical Wnt signaling and accelerated aging. Thus abnormally high, or low, C1q levels could possibly play a role in various disease states caused by increased, or decreased, Wnt signaling.

There is growing evidence that C1q binding, leading to activation of C1r and C1s, may trigger other functions *via* activated C1s, unexpectedly, cleaving nuclear antigens, MHC class I antigens and other proteins, as well as lipoprotein receptor 6 (45), thus opening up many other biological pathways, besides complement, that may be triggered by C1q binding.

A recent study has indicated that non-bone marrow derived, locally synthesized, C1q may play a role in enhancing tumor progression by facilitating cancer cell seeding and promoting angiogenesis (46). This view is consistent with the finding of increased expression of the genes of the chains of C1q correlating with a poor prognosis in breast cancers (47). However, other studies indicate that C1q may possibly play a protective role, as judged by C1q-enhanced apoptosis in an ovarian cancer cell line (48) and activation of a tumor suppressor to induce apoptosis in prostate cancer cells (49).

## Conclusion

There have been many publications concerned with the possible functions mediated *via* C1q, since its first description in 1961 (5), and perhaps more the use of the well-defined, globular, and collagen-like, fragments of C1q, monoclonal antibodies against these regions, or mutation of specific residues within the expressed whole recombinant molecule (4), will aid understanding of how exactly C1q is interacting with the large number of targets (in the region of 100, to date) to which it has been reported to bind. The use of a short synthetic triple helical peptide, corresponding to the binding site on C1q for the C1r_2_–C1s_2_ complex (22), to define the precise region on C1q involved in that interaction shows there are possibly many other innovative ways, utilizing protein fragments or synthetic peptides, which will allow the exploration, at the molecular level, of the functions of this versatile molecule.

## Author Contributions

The author confirms being the sole contributor of this work and approved it for publication.

## Conflict of Interest Statement

The author declares that the research was conducted in the absence of any commercial or financial relationships that could be construed as a potential conflict of interest.

## Abbreviations

CTRP, C1q/TNF-related protein; MBL, mannose binding lectin; MASP, MBL-associated serine protease; CRP, C-reactive protein; CUB, complement C1r/C1s; EGF, epidermal growth factor.
